# Short- and Long-Term Visual Outcomes in Patients Receiving Intravitreal Injections: The Impact of the Coronavirus 2019 Disease (COVID-19)—Related Lockdown

**DOI:** 10.3390/jcm11082097

**Published:** 2022-04-08

**Authors:** Vivian Paraskevi Douglas, Konstantinos A. A. Douglas, Demetrios G. Vavvas, Joan W. Miller, John B. Miller

**Affiliations:** Retina Service, Massachusetts Eye and Ear, Department of Ophthalmology Harvard Medical School, Boston, MA 02114, USA; douglasvivianp@gmail.com (V.P.D.); douglaskonstantinos@gmail.com (K.A.A.D.); demetrios_vavvas@meei.harvard.edu (D.G.V.); joan_miller@meei.harvard.edu (J.W.M.)

**Keywords:** delay in care, anti-VEGF, nonadherence, nonpersistence, noncompliance, treatment discontinuation, pandemic

## Abstract

*Purpose*: To investigate the short- and long-term impact of COVID-19—related lockdown on the vision of patients requiring intravitreal injections (IVI) for neovascular Age-related Macular degeneration (nvAMD), diabetic retinopathy (DR), central retinal vein occlusion (CRVO), or branch retinal vein occlusion (BRVO). *Methods*: This is a retrospective study from the Retina department of three Mass Eye and Ear centers. Charts of patients age of ≥ 18 years with any of the abovementioned diagnoses who had a scheduled appointment anytime between 17 March 2020 until 18 May 2020 (lockdown period in Boston, Massachusetts) were reviewed at baseline (up to 12 weeks before the lockdown), at first available follow-up (=actual f/u) during or after the lockdown period, at 3 months, 6 months, and at last available completed appointment of 2020. *Results*: A total of 1001 patients met the inclusion criteria. Of those patients, 479 (47.9%) completed their intended f/u appointment, while 522 missed it (canceled and “no show”). The delay in care of those who missed it was 59.15 days [standard deviation (SD) ± 49.6]. In these patients, significant loss of vision was noted at actual f/u [Best corrected visual acuity (BCVA) in LogMAR (Logarithm of the Minimum Angle of Resolution)—mean (±SD)—completed: 0.45 (±0.46), missed: 0.53 (±0.55); *p* = 0.01], which was more prominent in the DR group [Visual acuity (VA) change in LogMAR—mean (±SD); completed: 0.04 (±0.28), missed: 0.18 (±0.44); *p* = 0.02] and CRVO [completed: −0.06 (±0.27), missed: 0.11 (±0.35); *p* = <0.001] groups followed by nvAMD [completed: 0.006 (±0.16), missed: 0.06 (±0.27); *p* = 0.004] and BRVO [completed: −0.02 (±0.1), missed: 0.03 (±0.14); *p* = 0.02] ones. Overall, a higher percent of people who missed their intended f/u experienced vision loss of more than 15 letters at last f/u compared to those who completed it [missed vs. completed; 13.4% vs. 7.4% in nvAMD (*p* = 0.72), 7.8% vs. 6.3% in DR (0.84), 15.5% vs. 9.9% in CRVO (*p* < 0.001) and 9.6% vs. 2% in BRVO (*p* = 0.48)]. *Conclusions*: Delay in care of about 8.45 weeks can lead to loss of vision in patients who receive IVI with DR and CRVO patients being more vulnerable in the short-term, whereas in the long-term, CRVO patients followed by the nvAMD patients demonstrating the least vision recovery. BRVO patients were less likely to be affected by the delay in care. Adherence to treatment is key for maintaining and improving visual outcomes in patients who require IVI.

## 1. Introduction

Intravitreal injections (IVI) are currently the most commonly performed ophthalmic in-office procedures used for treating a wide range of retinal diseases [1]. Anti-vascular endothelial growth factor (anti-VEGF) injections have been widely and effectively used for the treatment of neovascular AMD (nvAMD) [2,3], choroidal neovascularization (CNV) of other causes ([4,5] as well as macular edema secondary to diabetes [6,7] or retinal vein occlusion (RVO) [8,9,10]. The frequency of administration of these medications is typically every 4 to 8 weeks, and it largely depends on the underlying pathology as well as the response to treatment [11].

Studies have demonstrated that in clinical practice, patients with nvAMD, diabetic macular edema (DME), and RVO have inferior visual outcomes compared to the results from randomized clinical trials, likely due to less adherence [12,13]. Factors that contribute to non-adherence [or else known as non-compliance [14] or non-attendance [15] such as health system-related factors (e.g., low clinic capacity for scheduling a visit within the time frame as suggested by the provider, patient illness, socioeconomic factors) and nonpersistence (also known as treatment discontinuation/cessation [16]) such as worse baseline VA and poor response to treatment represent a significant barrier for optimization of real-world visual outcomes in patients receiving IVI therapy [14].

In December 2019, severe acute respiratory syndrome coronavirus 2 (SARS-CoV-2) was first reported in China in the city of Wuhan. On 11 March 2020, the World Health Organization (WHO) declared the rapidly spreading of this novel coronavirus outbreak a public health emergency of international concern (PHEIC) [17]. In response to the COVID-19 pandemic, a stay-at-home advisory was announced on 15 March by the Governor of Massachusetts, which initially took effect from 24 March until 7 April and later extended until 18 May [18]. In addition, the American Academy of Ophthalmology recommended that ophthalmologists should only provide urgent or emergent clinical and surgical care as determined by each provider’s clinical judgment [19].

In this study, we sought to analyze the impact of the COVID-19 and its related lockdown on the visual outcomes in patients requiring IVI as part of their treatment regimen for nvAMD, DR, CRVO, or BRVO at their first completed f/u and at subsequent visits over longer time periods.

## 2. Methods

### 2.1. Data Collection

In this retrospective study, all data were collected and analyzed in accordance with the policies and procedures of the Institutional Review Board of the Massachusetts Eye and Ear (MEE) and Partners Healthcare (Protection of Human Subjects). Due to the retrospective nature of this study, written informed consent was not required.

We collected data from the Retina Department of 3 MEE eye centers (Main Campus, Longwood, Stoneham) between 1 December 2019 to 31 December 2020. Electronic charts of patients who had a scheduled visit anytime between 17 March 2020 until 18 May 2020 (COVID-19 lockdown period in Massachusetts) were reviewed.

In this cohort, patients ≥ 18 years old who had received an injection (anti-VEGF) within a 12-week period prior to 17 March 2020 and also had a diagnosis of nvAMD, DR, CRVO, or BRVO were included in the study, and their charts were reviewed at baseline, at first available follow-up (during or after lockdown period), at 3-months (10–14 weeks), 6-months (22–26 weeks), and at the last available completed appointment of 2020 [mean (±SD): 35.1 (±7.16) weeks]. Patients who did not need an injection or had received an injection more than 12 weeks prior to 17 March 2020 were not included in the analysis.

First available scheduling status [=Intended follow up (f/u) time] during the COVID-19-related lockdown was collected for all patients and further categorized as completed if the patient was seen by a provider and missed if either the patient or the provider/institution canceled the visit (canceled) or did not present to the eye center or prior notified the team (“no show”).

The first completed appointment during or outside the lockdown period was documented, and the actual f/u time was calculated. The difference of actual f/u and intended f/u time was also calculated and represented the delay in receiving treatment according to the provider’s suggestion. As such, if a patient had completed the intended f/u time, the actual f/u time was the same as the intended one, and there were no delays in the care of these patients.

Best corrected visual acuity (BCVA) of all treated eyes and their fellow eyes was measured using Early Treatment Diabetic Retinopathy Study (ETDRS) or Snellen eye chart and converted to the logarithm of the minimum angle resolution (LogMAR) [20]. A LogMAR change of 0.12 equals approximately the loss of 6 letters [21].

Five (5) groups of vision change in letters (+6–+15, ≥+15, −5–+5, −6–+15, ≤−15) were created in order to further investigate and compare the percentage of people (completed vs. missed intended f/u) who either gained, lost, or maintained (±5) their vision at first (actual f/u) and last f/u.

Patients who had not scheduled or had not completed a scheduled appointment during or after the lockdown period were considered as “lost to follow-up” patients. Data of those patients were collected and analyzed as well.

### 2.2. Statistical Analysis

The statistical analysis was performed using SPSS software version 28 (SPSS, Chicago, IL, USA). The Shapiro–Wilk test was used to examine normal distributions for all variables. Relationships between categorical variables were assessed using Fisher’s exact and Chi-Square tests. Relationships between continuous variables were assessed using independent samples *t*-test and ANOVA. Linear regression analysis was performed in order to evaluate the relationship between vision change and delays in care. *p* values <0.05 were considered statistically significant.

## 3. Results

Of a total of 1970 adult patients with a diagnosis of either nvAMD, DR, CRVO, or BRVO, 1001 patients met the inclusion criteria. Of these patients, 479 (47.9%) completed their intended follow-up appointment, and 522 (52.1%) missed it. 

Patients of age less than 67 and greater than 87 years were more likely to cancel or no show up to their scheduled appointment compared to those who belonged to the 67−76 or 77−87 groups (completed vs. missed: 43.2% vs. 56.8%; 39.4% vs. 60.6%; 51.2% vs. 48.8%, 52.3% vs. 47.7%, respectively; *p =* 0.01) (Table 1).

With regards to the diagnosis, while there were no significant differences in completed and missed appointments in patients with nvAMD (completed: 51.1%, missed: 48.9%; *p =* 0.6), CRVO (completed: 48.2%, missed: 51.8%; *p =* 0.7), and BRVO (completed: 49%, missed: 51%; *p =* 0.84), patients with DR had higher numbers of missed appointments (completed: 38%, missed: 62%; *p* < 0.001) (Table 1).

In our cohort, significant differences among races/ethnicities were observed with Blacks or African Americans having a significantly higher number of missed appointments compared to Whites or Caucasians, Asians, and “Other” (Hispanic or Latino, American Indian or Alaska Native, Declined, Unavailable) [completed vs. missed: 33.9% vs. 66.1%, 50% vs. 50%, 36.6% vs. 63.4%, 46.5% vs. 53.5%, respectively; *p =* 0.038] (Table 1).

Furthermore, scheduling status differed significantly among the three locations (*p* < 0.001) with more missed appointments being reported in one of the regional eye centers located approximately 4 miles away from the main campus (completed: 29.6%, missed: 70.4%; *p* < 0.001), followed by the main campus (completed: 46.5%, missed: 53.5%; *p =* 0.13). In the regional center located approximately 10 miles away from the main campus, the highest number of completed appointments was documented (completed: 53.3%, missed: 46.7%; *p =* 0.17). (Table 1)

On average, the intended f/u time was 50.61 (±20.6) days (*p =* 0.234) and the actual f/u time was 83.77 (SD: ±53.7) days with patients with DR having significantly longer f/u intervals compared to the nvAMD, CRVO and BRVO patients [93.12 (±61.1), 77.65 (±47.5), 81.13 (±52.9), 83.21 (±53.3), respectively; *p =* 0.003]. The average delay in care was 59.15 (SD: ±49.6) days with longer delays in care reported in the DR group and shortest in the nvAMD group [DR: 69.6 (±39.4), nvAMD: 52.62 (±30.1), CRVO: 58.4 (±32.2), BRVO: 59.5 (±32.3); *p* < 0.001] (Supplementary Table S1).

**Figure 1:**
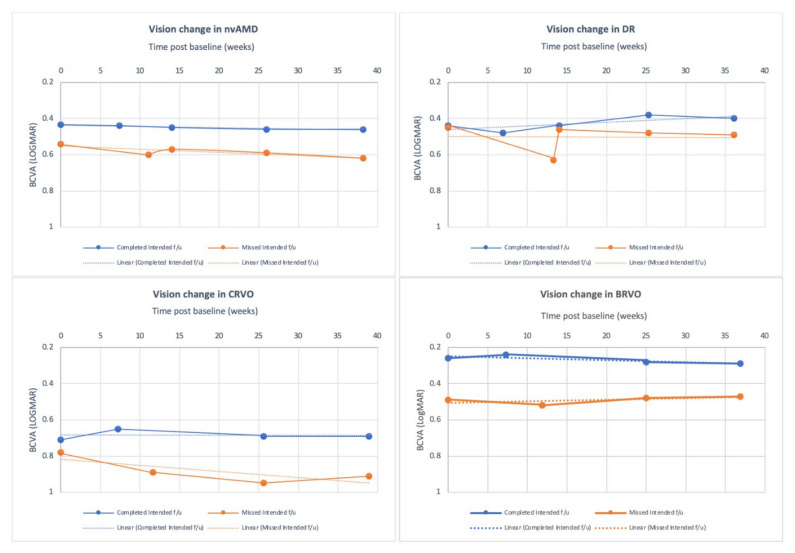
BCVA (logMAR) at actual f/u, at 3-month (only nvAMD, DR), 6-month and at last f/u (completed vs. missed intended f/u) by diagnosis. Abbreviations: nvAMD: neovascular Age-related Macular Degeneration; DR: diabetic retinopathy; CRVO: central retinal vein occlusion; BRVO: branch retinal vein occlusion; BCVA: best corrected visual acuity; f/u: follow up; LogMAR: Logarithm of the Minimum Angle of Resolution. LogMAR of (+) 0.1 equals loss of 5 letters.

BCVA at actual and last f/u of those who completed the intended f/u were similar but significantly differed from the BCVA of those who missed it [0.44 ± 0.45 vs. 0.53 ± 0.55; *p =* 0.01 (actual f/u), 0.45 ± 0.46 vs. 0.6 ± 0.61; *p* < 0.0001 (last f/u)] (Table 1, Figure 1).

Upon analysis of the reason for cancelation, more appointments were canceled by the patients rather than the provider-institution [311 (65.5%) vs. 164 (34.5%)]. There was no impact on the delay in care, in VA at actual and at last f/u in any of the four diagnoses (Supplementary Table S2).

Furthermore, in a sub-analysis of those who had less than 6 weeks delay in care versus those who had more than 12 weeks delay in care showed that the average VA change at actual and last f/u significantly differed with patients at the >12 weeks delay group losing on average 8 letters at both time frames while those at <6 weeks group losing 1.5–2 letters [VA change (LogMAR) <6 weeks vs. >12 weeks: actual f/u—0.03 (±0.23) vs. 0.16 (±0.45); *p* < 0.001, last f/u: 0.04 (±0.33) vs. 0.16 (±0.52); *p =* 0.003] (Supplementary Table S3).

### 3.1. Neovascular AMD (nvAMD)

In the nvAMD group, patients of age greater than 87 years had more missed appointments than the other age groups [completed vs. missed: 46.5% vs. 53.5% (<67 years), 56.5% vs. 43.5% (67–76), 54.5% vs. 45.5% (77–87), 40.3% vs. 59.7% (>87); *p =* 0.023]. Intended f/u time was similar in both completed and missed groups (51.89 ± 21.1, 51.86 ± 22.9 days; *p =* 0.99) while the actual f/u was significantly longer in those who missed the intended f/u (51.89 ± 21.1, 104.5 ± 52.6 days; *p* < 0.001). 

**Figure 2:**
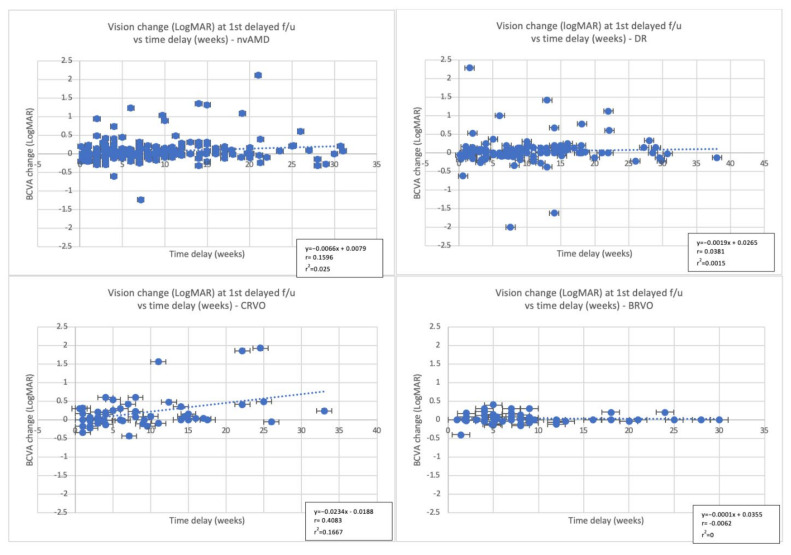
Regression analysis of the BCVA change (LogMAR) at actual f/u in patients who missed their intended f/u and time delay in weeks. Abbreviations: nvAMD: neovascular Age-related Macular Degeneration; DR: diabetic retinopathy; CRVO: central retinal vein occlusion; BRVO: branch retinal vein occlusion; BCVA: best corrected visual acuity; f/u: follow up; r = correlation coefficient; LogMAR: Logarithm of the Minimum Angle of Resolution. LogMAR of (+) 0.1 equals loss of 5 letters.

Statistically significant changes were noted in the baseline BCVA in both the injected and fellow eyes [completed vs. missed: 0.44 ± 0.41 vs. 0.54 ± 0.53; *p =* 0.005, 0.54 ± 0.67 vs. 0.69 ± 0.79; *p* < 0.001, respectively). Moreover, VA change in the injected eyes at actual and last available f/u was also significantly different with patients who completed their intended f/u losing less vision than those who missed it (actual f/u: 0.006 ± 0.16 vs. 0.06 ± 0.27; *p =* 0.004, last f/u: 0.02 ± 0.24 vs. 0.08 ± 0.36; *p =* 0.023). (Table 2) A very weak positive linear relationship between VA change at actual f/u and time delay (weeks) was noted in these patients (r^2^ = 0.025, F (1281) = 7.28, *p =* 0.007, Figure 2).

**Figure 3:**
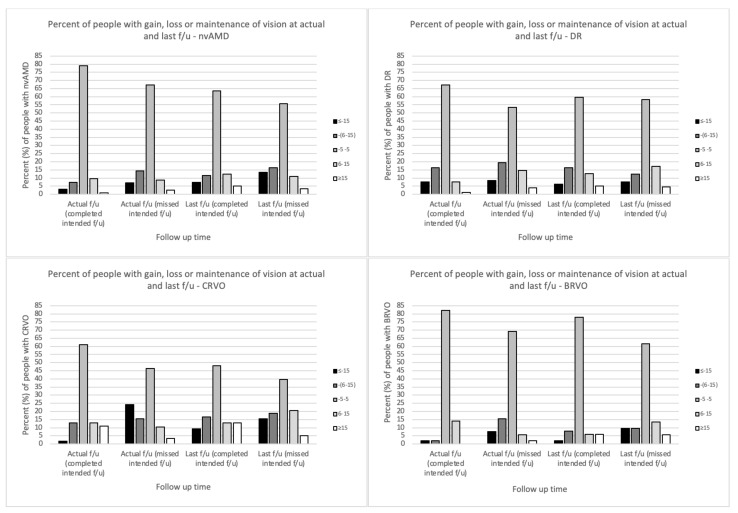
Bar chart depicting the percent of people (completed vs. missed intended f/u) who either maintained, gained, or lost vision (letters) at the intended f/u and last f/u. **Abbreviations**: nvAMD: neovascular Age-related Macular Degeneration; DR: diabetic retinopathy; CRVO: central retinal vein occlusion; BRVO: branch retinal vein occlusion; BCVA: best corrected visual acuity; f/u: follow up; %: percent; LogMAR: Logarithm of the Minimum Angle of Resolution. LogMAR of (+) 0.1 equals loss of 5 letters.

With regards to letter gain or loss, 79.1% of patients who completed vs. 67.1% of those who missed the intended f/u had vision change of a range of −5 to +5 letters at actual f/u whereas 3% vs. 7.1% lost more 15 letters (*p =* 0.23). At last f/u, this loss was increased to 7.4% for the ones who completed vs. 13.4% for those who missed it with 5.1% vs. 3.5% gaining >15 letters, respectively (*p =* 0.48, Figure 3, Supplementary Table S4).

### 3.2. Diabetic Retinopathy (DR)

In the DR group, the intended f/u time was comparable between completed and missed groups (47.9 ± 19.6, 48.6 ± 22.2 days; *p =* 0.84), whereas the actual f/u was significantly longer in those who missed the intended f/u (51.89 ± 21.1, 118.2 ± 61.4 days; *p* < 0.001).

Greater VA change was noted at actual f/u in the injected eyes in those who missed their f/u (completed: 0.04 ± 0.28 vs. missed: 0.18 ± 0.44; *p =* 0.02) and at baseline in the fellow eyes (0.58 ± 0.76 vs. 0.63 ± 0.82; *p* < 0.001). (Table 3) Similar to the nvAMD group, a very weak positive linear relationship between VA change at actual f/u and time delay (weeks) was noted (r^2^ = 0.0015, F (1127) = 0.19, *p =* 0.66, Figure 2).

Overall, more DR patients who missed their visit lost 6 or more letters at actual f/u compared to those who completed it (27.9% vs. 24.1%, *p =* 0.25) whereas at last f/u more were able to gain 6 or more letters (21.8% vs. 17.8%, *p =* 0.84). (Figure 3, Supplementary Table S4).

### 3.3. Central Retinal Vein Occlusion (CRVO)

The actual f/u time in CRVO patients who missed their intended f/u was notably prolonged compared to those who completed it (111 ± 57.3 vs. 49.1 ± 17.4 days, respectively; *p* < 0.001). Statistically significant VA changes at actual f/u (completed: −0.06 ± 0.27 vs. missed: 0.11 ± 0.35; *p* < 0.001), at 6 months (−0.02 ± 0.34 vs. 0.17 ± 0.55; *p =* 0.038) as well as in baseline BCVA in the fellow eyes (0.20 ± 0.52 vs. 0.32 ± 0.78; *p* < 0.001) were demonstrated in the CRVO patients who missed their intended f/u. (Table 4) There was a moderate positive linear relationship between time delay (weeks) and VA change at actual f/u (r^2^ = 0.17, F(1,56) = 11.2, *p =* 0.001, Figure 2).

A significant percent of CRVO people in the missed group lost more than 15 letters at actual f/u [24.1% vs. 19% (completed); *p* < 0.0001] while at last f/u this difference was less prominent (9.3% vs. 15.5%; *p =* 0.1). At last f/u both groups had similar odds of gaining >6 letters [26% (completed) vs. 25.9% (missed); *p =* 0.1]. (Figure 3, Supplementary Table S4).

### 3.4. Branch Retinal Vein Occlusion (BRVO)

In the BRVO group, baseline BCVA in both the injected (0.26 ± 0.25 vs. 0.49 ± 0.53; *p =* 0.005) and fellow eyes (0.26 ± 0.59 vs. 0.23 ± 0.43; *p* < 0.001) was significantly lower. The VA change at actual f/u (−0.02 ± 0.10 vs. 0.03 ± 0.14; *p =* 0.02) and last BCVA 0.29 ± 0.35 vs. 0.46 ± 0.44; *p =* 0.014) were also significantly different among patients who completed and those who missed the intended f/u. The average actual f/u time in the missed group was 110.1 ± 58.6 days, which was remarkably longer than that seen in the completed one (52.4 ± 19.4; *p* < 0.001) (Table 5). A very weak positive linear relationship of VA change over time was observed in BRVO patients (r^2^ < 0.001, F(1,50) = 0.0019, *p =* 0.965, Figure 2).

At actual f/u and at last f/u, the patients who completed the f/u either had significantly better visual outcomes (maintained or gained vision) compared to those who missed it (actual f/u: 96% vs. 76.9%; *p =* <0.001, last f/u: 90% vs. 80.8%; *p =* 0.04, Figure 3, Supplementary Table S4).

A total of 94 patients never completed an appointment (“lost to follow-up”) during or after the lockdown period. Of those, 85 (90.4%) patients canceled their appointment and 9 (9.6%) were “no show.” A comparison of the demographic, as well as the clinical characteristics of this group and the entire cohort, was performed. Overall, there were no statistically significant differences with regards to age (total and by group), gender, race, diagnosis, and reason for cancellation. However, in the “lost to follow-up” group, the BCVA of both the injected and fellow eyes was significantly worse compared to the average BCVA of the entire cohort [injected: 0.69 (±0.56) vs. 0.49 (±0.5); *p =* <0.001, fellow: 0.72 (±0.84) vs. 0.53 (±0.73); *p =* 0.017, respectively]. (Supplementary Table S5).

## 4. Discussion

In this retrospective study we examined the short- and long-term visual outcomes in patients with a diagnosis of nvAMD, DR, CRVO, or BRVO who have been receiving IVI as part of their treatment regimen and also had a scheduled appointment during the period of lockdown in Boston (MA). The average delay in care was 59.15 days (8.45 weeks). At actual f/u, vision maintenance was noted in patients who completed the f/u while loss was demonstrated in those who missed it (canceled + “no show”) and this was more pronounced in DR and CRVO patients. At last f/u, CRVO and nvAMD patients who missed their visit were less likely to experience a recovery of vision. There was no strong relationship in VA change in those who missed their f/u and the time delay in their care.

In our study, older patients and minorities were more likely to cancel or no show up to their appointment. It has been well established that racial/ethnic minority groups and elder patients have higher lapses in care [22,23] and are disproportionally affected by COVID-19, and as such these findings also support and reflect the observations of other studies [24]. In the study of Ehlken et al., it was shown that older patients with nvAMD receiving anti-VEGF and those with lower baseline VA were at higher risk of non-adherence to f/u compared to BRVO and DR patients [12], a finding that was also noted in our study.

Distance-to-facility and lack of transport are known barriers for non-adherence and nonpersistence to treatment [14,25,26]. In this study, the highest number of completed visits was reported in Stoneham, a town located outside Boston city. Even though distances between patient’s and hospital’s zone improvement plan (ZIP) codes were not calculated, we assumed that patients were more likely to complete a scheduled appointment in an eye center that was near their home due to several reasons such as transportation issues (less costs, travel time, caregiver availability), less waiting time at clinic and more flexible clinic time schedules.

As opposed to the findings from randomized controlled clinical trials, studies from real world settings have demonstrated that treatment interruption is oftentimes associated with suboptimal visual outcomes with baseline BCVA, injection frequency, age, and comorbidities playing an important role [12,27,28,29,30]. High rates of visit non-adherence have been reported in people with poor visual acuity in untreated eyes [31]. In our cohort, significant BCVA changes at baseline among completed and missed groups in injected eyes were found in nvAMD and BRVO groups but also in all groups in the fellow eyes but not in DR and CRVO ones.

This is one of the few studies where the effect of short lapses in care on vision are examined. We additionally investigated their effect over a longer period of time for each diagnosis and compared the visual outcomes between those who had <6 weeks delay to those with >12 weeks delay in care. The average delay in care in our cohort was 59.15 days (8.45 weeks). In the study of Rahimzadeh et al., there was no significant vision loss in nvAMD and DR patients who had an average delay in care of 47 (range 15–166) days and 56 (17–207) days, respectively, and similar to our findings, there was no correlation between duration of delay and VA change [32]. However, in contrast to our study, they included only new nvAMD and DR patients who received the same anti-VEGF agent (aflibercept) between January 1st to July 15th 2020 and the delays in care were shorter by approximately 5 days for the nvAMD and 14 days for DR patients compared to those seen in our study. Moreover, in the study of Song et al., where the average delay in care was 5.34 weeks, it was shown that DR and RVO patients were at higher risk of vision loss even with short delays in care [33]. In our study, DR patients were also susceptible to vision loss in the short-term, whereas in the long-term most people recovered well. In contrast to their study, we examined CRVO and BRVO patients separately and followed all groups over longer periods. We noticed that CRVO patients were more vulnerable to vision loss in both short- and long-term while BRVO patients were the least to be affected by the lapses in care.

Visual outcomes in patients with nvAMD, DR and RVO with a delay of more than 2 weeks were evaluated by Naravane et al. [34]. The overall VA difference of pre- and post-lockdown BCVA in LogMAR was 0.136 in the delayed group and 0.008 in the control (no delays) while in our study there were lower at an average of 0.03 and −0.01, respectively. (Table 1) Nonetheless, while in their subanalysis the VA changes in the DR group were similar to our results, those in the nvAMD patients were worse in both the delayed and control groups (delayed: 0.125 vs. 0.06, not delayed: 0.028 vs. 0.006). In the same study, no significant differences were reported in the RVO group as opposed to our findings. The study team, however, acknowledged the small sample size as an important limitation, which was more prominent in the RVO group [34]. In the study of Stone et al., a greater percent of DR patients in the delayed group had their vision maintained (±5 letters) at f/u (mean delay: 13.1 weeks) when compared to nvAMD and RVO groups (~62% in both) and approximately a 10% of people in all 3 groups lost >15 letters [35]. We observed that the BRVO (69.2%) followed by nvAMD (67.1%) patients were more likely to experience vision maintenance at actual f/u compared to DR (53.5%) and CRVO (46.6%) ones. The same team also evaluated the percentage of eyes whose VA returned back to baseline since the delayed visit (range: January 2020 until November 2020) but at any point. They noted that 89.7% of DR, 76.9% of RVO and 74.6% of nvAMD affected eyes had their vision returned to baseline at some point. In our cohort, CRVO patients had the lowest number of vision maintenance (39.7%) compared to their baseline VA followed by nvAMD (55.8%), DR (58.1%) and BRVO (61.5%). The smaller sample size of their study, the grouping of RVO cases along with the fact that they included VA maintenance cases at any point since actual f/u might have contributed to the abovementioned discrepancies compared to our findings. It should be also emphasized that in this study we included only established patients and not new ones. It is thus, possible that some patients may have been receiving treatment for a long time and this can explain the relative stability seen in the VA at actual f/u in some patients who completed their intended f/u, which have also been described in previous studies [36]. Lastly, we believe that concurrent presence of additional ocular findings (e.g., vitreous or subretinal hemorrhages, neovascular glaucoma, etc.) is likely associated with less favorable outcomes in the short- and long-term. In this study we did not specifically account for their impact on the visual acuity, however, we investigated the long-term outcomes in patients who had missed their intended f/u and also presented with severe visual loss at actual f/u. Our findings suggest that the original vision loss from delayed care was not a permanent one for at least 30% of these patients but was for 70% and for each disease these proportions were different.”

Among the strengths of our study was that we followed the same cohort at close intervals over a long period. For our analysis we used real-world data where clinical trial rules were not applied to any patient or group. To our knowledge, this is the first study where comprehensive data across different race/ethnic groups and diseases are also provided and the importance of incorporating equal representation of racial/ethnic groups in future studies is also stressed.

As with the majority of the studies, the results reported also in this study have to be seen in light of some limitations. This is a retrospective study where associated ocular conditions, duration of disease, type of anti-VEGF factor, total number of injections as well as other non-ocular related health issues were not accounted for during the analysis, which could probably have led to confounding results.

During the COVID-19 crisis, several preventative measures, as well as novel methods, were applied in an effort to promote adherence and persistence to care. Beyond the measures implemented as per global and national guidelines, in our department during the reminder phone calls, dedicated time for addressing questions and fears of being contaminated during their visit was offered, personalized recommendations were made, up-to-date safety information on Mass Eye and Ear website and phone center was provided as well as tele-consultations. Additionally, in order to reduce the burden, there was a prioritization of ophthalmic outpatient appointments where patients at increased risk for visual loss were allowed to complete an in-person visit.

## 5. Conclusions

In conclusion, this study demonstrates that delays in care can lead to significant vision loss in nvAMD, DR, CRVO, and BRVO patients on IVI with DR and CRVO patients losing more vision than nvAMD and BRVO ones in the short-term. In the long-term, patients with CRVO are less prone to the recovery of vision compared to nvAMD and DR, whereas BRVO patients are more likely to maintain or even gain vision. Higher rates in visit adherence were noted in rural eye centers likely reflecting the multifaceted nature of non-adherence and nonpersistence to treatment. Based on the findings of this study, a treatment paradigm shift in patients receiving IVI in conjunction with the development and implementation of measures that would promote adherence (e.g., effective communication, patient education etc.), can lead to superior visual outcomes in short- and long-term.

## Figures and Tables

**Table 1:** Demographic characteristics of the cohort based on the intended f/u status [Completed, Missed (Canceled + “no show”)].

Intended f/u Status	Completed (*n* = 479)	Missed (Canceled + “No Show”) *n* = 522	Total (*n* = 1001)	*p* Value
**Diagnosis *n* (%)**				**0.01**
nvAMD	296 (61.8)	283 (54.2)	579 (57.8)	
BRVO	50 (10.4)	52 (10)	102 (10.2)	
CRVO	54 (11.3)	58 (11.1)	112 (11.2)	
DR	79 (16.5)	129 (24.7)	208 (20.8)	
**Race *n* (%)**				**0.038**
White or Caucasian	364 (76)	364 (69.7)	728 (72.7)	
Asian	15 (3.1)	26 (5)	41 (4.1)	
Black or African American	21 (4.4)	41 (7.9)	62 (6.2)	
Other	79 (16.5)	91 (17.4)	170 (17)	
**Gender *n* (%)**				0.62
Female	278 (58)	295 (56.5)	573 (57.2)	
Male	201 (42)	227 (43.5)	428 (42.8)	
**Age in years *n* (%)**				**0.017**
Less than 67	111 (23.2)	146 (28)	257 (25.7)	
67–76	145 (30.3)	138 (26.4)	283 (28.3)	
77–87	167 (34.9)	152 (29.1)	319 (31.9)	
Greater than 87	56 (11.7)	86 (16.5)	142 (14.2)	
**Mean age (±SD)**	74.1 (11.9)	74.1 (12.5)	74.1 (12.4)	0.9
**Location *n* (%)**				**<0.0001**
Main Campus	216 (45.1)	248 (47.5)	464 (46.4)	
Stoneham	234 (48.9)	205 (39.3)	439 (43.9)	
Longwood	29 (6)	69 (13.2)	98 (9.8)	
**Baseline BCVA [LogMAR; mean (±SD)]**	0.45 (0.46)	0.5 (0.5)	0.49 (0.51)	0.09
**BCVA at actual f/u [LogMAR; mean (±SD)]**	0.44 (0.45)	0.53 (0.55)	0.54 (0.55)	**0.01**
**BCVA at last f/u [LogMAR; mean (±SD)]**	0.45 (0.46)	0.6 (0.61)	0.53 (0.55)	**<0.0001**

Abbreviations: nvAMD: neovascular AMD; DR: diabetic retinopathy; CRVO: central retinal vein occlusion; BRVO: branch retinal vein occlusion; BCVA: best corrected visual acuity; f/u: follow up; *n* = number; SD: standard deviation; LogMAR: Logarithm of the Minimum Angle of Resolution.

**Table 2:** Demographic and clinical characteristics of nvAMD patients.

nvAMD	nvAMD (*n* = 579)	Completed Intended f/u (*n* = 296)	Missed Intended f/u (*n* = 283)	*p* Value
**Age in years *n* (%)**				**0.023**
Less than 67	43 (7.4)	20 (6.8)	23 (8.1)	
67–76	147 (25.4)	83 (28)	64 (22.6)	
77–87	255 (44.0)	139 (47)	116 (41)	
Greater than 87	134 (23.1)	54 (18.2)	80 (28.3)	
**Mean age (±SD)**	80.1 (8.9)	79.5 (8.5)	80.8 (9.3)	0.08
**Gender *n* (%)**				0.7
Female	374 (64.6)	189 (63.9)	185 (65.4)	
Male	205 (35.4)	107 (36.1)	98 (34.6)	
**Race *n* (%)**				0.11
White or Caucasian	476 (82.2)	249 (84.1)	227 (80.2)	
Asian	26 (4.5)	8 (2.7)	18 (6.4)	
Black or African American	7 (1.2)	2 (0.7)	5 (1.8)	
Hispanic or Latino	0 (0)	0 (0)	0 (0)	
Other	70	37 (12.5)	33 (11.6)	
**Location *n* (%)**				
Main Campus	255 (44.0)	126 (42.6)	129 (45.6)	**0.0016**
Stoneham	283 (48.9)	159 (53.7)	124 (43.8)	
Longwood	41 (7.1)	11 (3.7)	30 (10.6)	
**Intended f/u Time [Days, mean (SD)]**	51.88 (±22.00)	51.89 (±21.1)	51.86 (±22.9)	0.99
**Actual f/u Time [Days, mean (SD)]**	77.65 (±47.53)	51.89 (±21.1)	104.5 (±52.6)	**<0.001**
**Delays in care [Days, mean (SD)]**	52.62 (±30.1)	0	52.62 (±30.1)	NA
**Visual acuity baseline and VA changes from baseline overtime [LogMAR; mean (±SD)]**				
**Baseline BCVA** (up to 12 weeks before MA lockdown)—**injected** eyes		0.44 (±0.41)	0.54 (±0.53)	**0.005**
**Baseline BCVA** (up to 12 weeks before MA lockdown)—**fellow** eyes		0.54 (±0.67)	0.69 (±0.79)	**<0.001**
**Actual f/u** VA change		0.006 (±0.16)	0.06 (±0.27)	**0.004**
**3 months** VA change		0.01 (±0.21)	0.03 (±0.23)	0.32
**6 months** VA change		0.02 (±0.25)	0.05 (±0.30)	0.12
**Last f/u** VA change		0.02 (±0.24)	0.08 (±0.36)	**0.023**
**Last BCVA**		0.46 (±0.45)	0.62 (±0.59)	**<0.001**
**Last f/u Time [Days, mean (SD)]**		263.4 (±48)	270.8 (±50.7)	0.07

Abbreviations: nvAMD: neovascular AMD; VA: visual acuity; BCVA: best corrected visual acuity; f/u: follow up; *n* = number; LogMAR: Logarithm of the Minimum Angle of Resolution; SD: standard deviation; NA: not applicable; Missed: canceled + “no show”; LogMAR of (+) 0.1 equals loss of 5 letters.

**Table 3:** Demographic and clinical characteristics of DR patients.

DR	DR (*n* = 208)	Completed Intended f/u (*n* = 79)	Missed Intended f/u (*n* = 129)	*p* Value
**Age in years *n* (%)**				
Less than 67	135 (64.9)	51 (64.5)	84 (65.1)	0.247
67–76	57 (27.4)	19 (24.1)	38 (29.5)	
77–87	16 (7.7)	9 (11.4)	7 (5.4)	
Greater than 87	0 (0.0)	0 (0)	0 (0)	
**Mean age (±SD)**	61.7 (11.5)	61.6 (12.1)	61.7 (10.9)	0.48
**Gender *n* (%)**				0.867
Female	88 (42.3)	34 (43)	54 (41.9)	
Male	120 (57.7)	45 (57)	75 (58.1)	
**Race *n* (%)**				
White or Caucasian	110 (52.9)	46 (58.2)	64 (49.6)	0.63
Asian	6 (2.9)	2 (2.6)	4 (3.1)	
Black or African American	36 (17.3)	11 (13.9))	25 (19.4)	
Other	56 (26.9)	20 (25.3)	36 (27.9)	
**Location *n* (%)**				0.84
Main Campus	104 (50.0)	39 (49.4)	65 (50.4)	
Stoneham	72 (34.6)	29 (36.7)	43 (33.3)	
Longwood	32 (15.4)	11 (13.9)	21 (16.3)	
**Intended f/u Time [Days, mean (SD)]**	48.33 (±21.26)	47.9 (±19.6)	48.6 (±22.2)	0.84
**Actual f/u Time [Days, mean (SD)]**	93.12 (±61.15)	47.9 (±19.6)	118.2 (±61.4)	**<0.001**
**Delays in care [Days, mean (SD)]**	69.6 (±39.4)	0	69.6 (±39.4)	NA
**Visual acuity baseline and VA changes from baseline overtime [LogMAR; mean (±SD)]**				
**Baseline BCVA** (up to 12 weeks before MA lockdown)—**injected** eyes		0.44 (±0.49)	0.45 (±0.46)	0.95
**Baseline BCVA** (up to 12 weeks before MA lockdown)—**fellow** eyes		0.58 (±0.76)	0.63 (±0.82)	**<0.001**
**Actual f/u** VA change		0.04 (±0.28)	0.18 (±0.44)	**0.02**
**3 months** VA change		0.00 (±0.30)	0.01 (±0.35)	0.82
**6 months** VA change		−0.06 (±0.36)	0.03 (±0.34)	0.06
**Last f/u** VA change		−0.04 (±0.34)	0.04 (±0.44)	0.19
**Last BCVA**		0.40 (±0.32)	0.49 (±0.56)	0.2
**Last f/u Time [Days, mean (SD)]**		243.6 (±72.2)	258.6 (±54.6)	0.09

Abbreviations: DR: diabetic retinopathy; VA: visual acuity; BCVA: best corrected visual acuity; f/u: follow up; *n* = number; LogMAR: Logarithm of the Minimum Angle of Resolution; SD: standard deviation; NA: not applicable; Missed: canceled + “no show”; LogMAR of (+) 0.1 equals loss of 5 letters.

**Table 4:** Demographic and clinical characteristics of CRVO patients.

CRVO	CRVO (*n* = 112)	Completed Intended f/u (*n* = 54)	Missed Intended f/u (*n* = 58)	*p* Value
**Age in years *n* (%)**				0.41
Less than 67	47 (42.0)	22 (40.7)	25 (43.1)	
67–76	38 (33.9)	21 (38.9)	17 (29.3)	
77–87	26 (23.2)	10 (18.5)	16 (27.6)	
Greater than 87	1 (0.9)	1 (1.9)	0 (0)	
**Mean age (±SD)**	68.3 (11.1)	68 (11.7)	69 (10.8)	0.32
**Gender *n* (%)**				0.98
Female	58 (51.8)	28 (51.9)	30 (51.7)	
Male	54 (48.2)	26 (48.1)	28 (48.3)	
**Race *n* (%)**				0.62
White or Caucasian	78 (69.7)	39 (72.2)	39 (67.3)	
Asian	2 (1.8)	2 (3.7)	0 (0)	
Black or African American	7 (6.2)	2 (3.7)	5 (8.6)	
Other	25 (22.3)	11 (20.4)	14 (24.1)	
**Location *n* (%)**				0.4
Main Campus	58 (51.8)	28 (51.8)	30 (51.7)	
Stoneham	39 (34.8)	21 (38.9)	18 (31)	
Longwood	15 (13.4)	5 (9.3)	10 (17.3)	
**Intended f/u Time [Days, mean (SD)]**	50.76 (±19.58)	49.1 (±17.4)	52.4 (±21.4)	0.37
**Actual f/u Time [Days, mean (SD)]**	81.13 (±52.92)	49.1 (±17.4)	111 (±57.3)	**<0.001**
**Delays in care [Days, mean (SD)]**	58.4 (±32.2)	0	58.4 (±32.2)	NA
**Visual acuity baseline and VA changes from baseline overtime ([LogMAR; mean (±SD)]**				
**Baseline BCVA** (up to 12 weeks before MA lockdown)—**injected** eyes		0.71 (±0.65)	0.78 (±0.72)	0.61
**Baseline BCVA** (up to 12 weeks before MA lockdown)—**fellow** eyes		0.20 (±0.52)	0.32 (±0.78)	**<0.001**
**Actual f/u** VA change		−0.06 (±0.27)	0.11 (±0.35)	**<0.001**
**3 months** VA change		−0.06 (±0.27)	0.03 (±0.39)	0.21
**6 months** VA change		−0.02 (±0.34)	0.17 (±0.55)	**0.038**
**Last f/u** VA change		−0.02 (±0.33)	0.13 (±0.57)	0.1
**Last BCVA**		0.69 (±0.66)	0.91 (±0.88)	0.14
**Last f/u Time [Days, mean (SD)]**		277.1 (±32.7)	255.4 (±53.8)	**0.01**

Abbreviations: CRVO: central retinal vein occlusion; VA: visual acuity; BCVA: best corrected visual acuity; f/u: follow up; *n* = number; LogMAR: Logarithm of the Minimum Angle of Resolution; SD: standard deviation; NA: not applicable; Missed: canceled + “no show”; LogMAR of (+) 0.1 equals loss of 5 letters.

**Table 5:** Demographic and clinical characteristics of BRVO patients.

BRVO	BRVO (*n* = 102)	Completed Intended f/u (*n* = 50)	Missed Intended f/u (*n* = 52)	*p* Value
**Age in years *n* (%)**				0.17
Less than 67	32 (31.4)	18 (36)	14 (26.9)	
67–76	41 (40.2)	22 (44)	19 (36.5)	
77–87	22 (21.6)	9 (18)	13 (25)	
Greater than 87	7 (6.9)	1 (2)	6 (11.6)	
**Mean age (±SD)**	71.2 (10.4)	68.9 (9.3)	73.4 (11.1)	**0.02**
**Gender *n* (%)**				0.68
Female	53 (52.0)	27 (54)	26 (50)	
Male	49 (48.0)	23 (46)	26 (50)	
**Race *n* (%)**				0.84
White or Caucasian	64 (62.7)	30 (60)	34 (65.4)	
Asian	7 (6.9)	3 (6)	4 (7.7)	
Black or African American	12 (11.8)	6 (12)	6 (11.5)	
Other	19 (18.6)	11 (22)	8 (15.4)	
**Location *n* (%)**				0.126
Main Campus	47 (46.1)	23 (46)	24 (46.1)	
Stoneham	45 (44.1)	25 (50)	20 (38.5)	
Longwood	10 (9.8)	2 (4)	8 (15.4)	
**Intended f/u Time [Days, mean (SD)]**	51.46 (±19.72)	52.4 (±19.4)	50.6 (±20.1)	0.64
**Actual f/u Time [Days, mean (SD)]**	83.21 (±53.28)	52.4 (±19.4)	110.1 (±58.6)	**<0.001**
**Delays in care [Days, mean (SD)]**	59.5 (±32.3)	0	59.5 (±32.3)	NA
**Visual acuity baseline and VA changes from baseline overtime [LogMAR; mean (±SD)]**				
**Baseline BCVA** (up to 12 weeks before MA lockdown)—**injected** eyes		0.26 (±0.25)	0.49 (±0.53)	**0.005**
**Baseline BCVA** (up to 12 weeks before MA lockdown)—**fellow** eyes		0.26 (±0.59)	0.23 (±0.43)	**<0.001**
**Actual f/u** VA change		−0.02 (±0.10)	0.03 (±0.14)	**0.02**
**3 months** VA change		−0.01 (±0.11)	0.04 (±0.20)	0.19
**6 months** VA change		0.02 (±0.24)	−0.01 (±0.37)	0.59
**Last f/u** VA change		0.03 (±0.25)	−0.02 (±0.42)	0.41
**Last BCVA**		0.29 (±0.35)	0.46 (±0.44)	**0.014**
**Last f/u Time [Days, mean (SD)]**		268.3 (±60.6)	248.8 (±63.9)	0.12

Abbreviations: BRVO: branch retinal vein occlusion; VA: visual acuity; BCVA: best corrected visual acuity; f/u: follow up; *n* = number; LogMAR: Logarithm of the Minimum Angle of Resolution; SD: standard deviation; NA: not applicable; Missed: canceled + “no show”; LogMAR of (+) 0.1 equals loss of 5 letters.

## Data Availability

The data presented in this study are available on request from the corresponding author. The data are not publicly available due to their containing information that could compromise the privacy of research participants.

## Supplementary Materials

The following are available online at https://www.mdpi.com/article/10.3390/jcm11082097/s1, Figure S1: visual acuity change (LogMAR) at 1st delayed f/u and last f/u by time delay (weeks), Table S1: intended f/u, actual f/u and delays in care by diagnosis; Table S2: delays in care and visual acuity changes based on the reason for cancelation (patient vs. provider-institution) by diagnosis; Table S3: baseline BCVA and visual changes at actual and last f/u in patients who missed <6 weeks compared to those who missed >12 weeks; Table S4: percent of people with letter gain or loss at actual and last f/u (corresponding to Figure 3); Table S5: demographic and clinical characteristics of lost to f/u patients compared to the entire cohort
