# Supplementary material for: Short- and Long-Term Visual Outcomes in Patients Receiving Intravitreal Injections: The Impact of the Coronavirus 2019 Disease (COVID-19)—Related Lockdown
Source: J Clin Med. 2022 Apr 8;11(8):2097. doi: 10.3390/jcm11082097 (PMC9029849; doi:10.3390/jcm11082097)
Supplement: Supplementary file 1 [file jcm-11-02097-s001.zip › jcm-1601358-supplementary.pdf]

Supplemental Table S1 – Intended f/u, actual f/u and delays in care by diagnosis

	nAMD (n=579)	DR (n=208)	CRVO (n=112)	BRVO (n=102)	Total (n=1001)	p value
**Intended f/u Time [Days, mean (SD)]**	51.88 (22.00)	48.33 (21.3)	50.76 (19.6)	51.46 (19.7)	50.61 (20.6)	0.234
**Actual f/u Time [Days, mean (SD)]**	77.65 (47.5)	93.12 (61.1)	81.13 (52.9)	83.21 (53.3)	83.77 (53.7)	**0.003**
**Delays in care [Days, mean (SD)]**	52.64 (30.1)	69.6 (39.4)	58.4 (32.2)	59.5 (32.3)	59.15 (49.6)	**<0.001**

Abbreviations: nvAMD: neovascular Age-related Macular Degeneration; DR: diabetic retinopathy; CRVO: central retinal vein occlusion; BRVO: branch retinal vein occlusion; f/u: follow up; SD: standard deviation

Supplemental Table S2 – Delays in care and visual acuity changes based on the reason for cancelation (patient vs provider-institution) by diagnosis

Reason for cancelation	Patient	Provider-Institution	P value
**nvAMD**	**n= 189**	**n=74**	
Delay in care [Days, mean (SD)]	50.93 (40.9)	53.65 (47.8)	0.32
VA change at actual f/u (LogMAR)	0.05 (0.23)	0.06 (0.27)	0.36
VA change at last f/u (LogMAR)	0.07 (0.36)	0.08 (0.28)	0.33
**DR**	**n=56**	**n=55**	
Delay in care [Days, mean (SD)]	70.7 (58.4)	74.9 (50.8)	0.34
VA change at actual f/u (LogMAR)	0.02 (0.41)	0.03 (0.36)	0.42
VA change at last f/u (LogMAR)	-0.006 (0.35)	0.06 (0.53)	0.22
**CRVO**	**n=33**	**n=21**	
Delay in care [Days, mean (SD)]	67.3 (56.9)	46 (46.3)	0.08
VA change at actual f/u (LogMAR)	0.17 (0.41)	0.2 (0.45)	0.39
VA change at last f/u (LogMAR)	0.12 (0.62)	0.19 (0.49)	0.33
**BRVO**	**n=33**	**n=14**	
Delay in care [Days, mean (SD)]	55.9 (46.9)	58.1 (34.8)	0.44
VA change at actual f/u (LogMAR)	0.01 (0.13)	0.08 (0.16)	0.09
VA change at last f/u (LogMAR)	-0.02 (0.33)	-0.09 (0.48)	0.29

Abbreviations: nvAMD: neovascular Age-related Macular Degeneration; DR: diabetic retinopathy; CRVO: central retinal vein occlusion; BRVO: branch retinal vein occlusion; BCVA: best corrected visual acuity; f/u: follow up; LogMAR: Logarithm of the Minimum Angle of Resolution; SD: standard deviation

* LogMAR of (+) 0.1 equals loss of 5 letters

Supplemental Table S3 – Baseline BCVA and visual changes at actual and last f/u in patients who missed <6 weeks compared to those who missed >12 weeks

	**<6 weeks (n=235)**	**>12 weeks (n=119)**	**P value**
**Baseline BCVA in LogMAR (up to 12 weeks before MA lockdown) [mean (SD)] – injected eyes**	0.49 (0.51)	0.58 (0.58)	0.06
**Baseline BCVA in LogMAR (up to 12 weeks before MA lockdown) [mean (SD)] – fellow eyes**	0.5 (0.73)	0.65 (0.82)	**0.03**
**Actual f/u VA change [LogMAR, mean (SD)]**	0.03 (0.23)	0.16 (0.45)	**<0.001**
**Last f/u VA change [LogMAR, mean (SD)]**	0.04 (0.33)	0.16 (0.52)	**0.003**

Abbreviations: BCVA: best corrected visual acuity; f/u: follow up; LogMAR: Logarithm of the Minimum Angle of Resolution

* LogMAR of (+) 0.1 equals loss of 5 letters

Supplemental Table S4 – Percent of people with letter gain or loss at actual and last f/u (corresponding to Figure 3)

Letter loss or gain	Actual f/u (completed intended f/u)	Actual f/u (missed intended f/u)	Last f/u (completed intended f/u)	Last f/u (missed intended f/u)
**nvAMD**				
**≤-15**	3.0	7.1	7.4	13.4
**-(6-15)**	7.4	14.5	11.5	16.3
**-5 -5**	79.1	67.1	63.5	55.8
**6-15**	9.5	8.8	12.5	11.0
**≥15**	1.0	2.5	5.1	3.5
**DR**				
**≤-15**	7.6	8.5	6.3	7.8
**-(6-15)**	16.5	19.4	16.5	12.4
**-5 -5**	67.1	53.5	59.5	58.1
**6-15**	7.6	14.7	12.7	17.1
**≥15**	1.3	3.9	5.1	4.7
**CRVO**				
**≤-15**	1.9	24.1	9.3	15.5
**-(6-15)**	13.0	15.5	16.7	19.0
**-5-5**	61.1	46.6	48.1	39.7
**6-15**	13.0	10.3	13.0	20.7
**≥15**	11.1	3.4	13.0	5.2
**BRVO**				
**≤-15**	2.0	7.7	2.0	9.6
**-(6-15)**	2.0	15.4	8.0	9.6
**-5 -5**	82.0	69.2	78.0	61.5
**6-15**	14.0	5.8	6.0	13.5
**≥15**	0.0	1.9	6.0	5.8

Abbreviations: nvAMD: neovascular Age-related Macular Degeneration; DR: diabetic retinopathy; CRVO: central retinal vein occlusion; BRVO: branch retinal vein occlusion; f/u: follow up

Supplemental Table S5 – Demographic and clinical characteristics of lost to f/u patients compared to the entire cohort

	**Canceled (n=85)**	**"No show" (n=9)**	**Total (n=94)**	**Total # of pts meeting inclusion criteria (n=1001)**	**p value**
**Age mean (±SD)]**	74.78±14.27	74.88±13.54	75.67±14.38	74.07±12.47	0.99
**Age n (%)**					
Less than 67	20 (23.5%)	3 (33.3%)	23 (24.5%)	257 (25.7)	0.89
67-76	21 (24.7%)	1 (11.1%)	22 (23.4%)	283 (28.3)	0.62
77-87	24 (28.2%)	1 (11.1%)	25 (26.6%)	319 (31.9)	0.58
Great than 87	20 (23.5%)	4 (44.4%)	24 (25.5%)	142 (14.2)	0.16
**Gender (Male)**	39 (45.9%)	3 (33.3%)	42 (44.7%)	428 (42.8)	0.81
**Race**					
White or Caucasian	61 (71.8%)	4 (44.4%)	65 (69.1%)	728 (72.7)	0.53
Asian	7 (8.2%)	1 (11.1%)	8 (8.5%)	41 (4.1)	0.59
Black or African American	6 (7.1%)	3 (33.3%)	9 (9.6%)	62 (6.2)	0.52
Other	11 (12.9)	1 (11.2)	12 (12.8)	170 (17)	0.61
**Diagnosis**					
nvAMD	49 (57.6%)	4 (44.4%)	53 (56.4%)	579 (57.8)	0.84
DR	19 (22.4%)	3 (33.3%)	22 (23.4%)	208 (20.8)	0.77
CRVO	7 (8.2%)	0 (0%)	7 (7.4%)	112 (11.2)	0.75
BRVO	10 (11.8%)	2 (22.2%)	12 (12.8%)	102 (10.2)	0.78
**Reason for cancellation**					
Patient	59 (69.4%)	NA	59 (69.4%)	311 (65.5)*	0.57
Provider	18 (21.2%)	NA	18 (21.2%)	164 (34.5)*	0.26
Deceased	8 (9.4%)	NA	8 (9.4%)	NA	NA
**BCVA**					
Injected eye	0.66 (0.57)	0.71 (0.47)	0.69 (±0.56)	0.49 (±0.5)	**<0.001**
Fellow eye	0.69 (0.83)	0.87 (0.91)	0.72 (±0.84)	0.53 (±0.73)	**0.017**

*out of 475 (patients who canceled their scheduled f/u)

Abbreviations: nvAMD: neovascular Age-related Macular Degeneration; DR: diabetic retinopathy; CRVO: central retinal vein occlusion; BRVO: branch retinal vein occlusion; BCVA: best corrected visual acuity; f/u: follow up; LogMAR: Logarithm of the Minimum Angle of Resolution; SD: standard deviation

* LogMAR of (+) 0.1 equals loss of 5 letters

Supplemental Figure S1 - Visual acuity change (LogMAR) at 1st delayed f/u and last f/u by time delay (weeks)

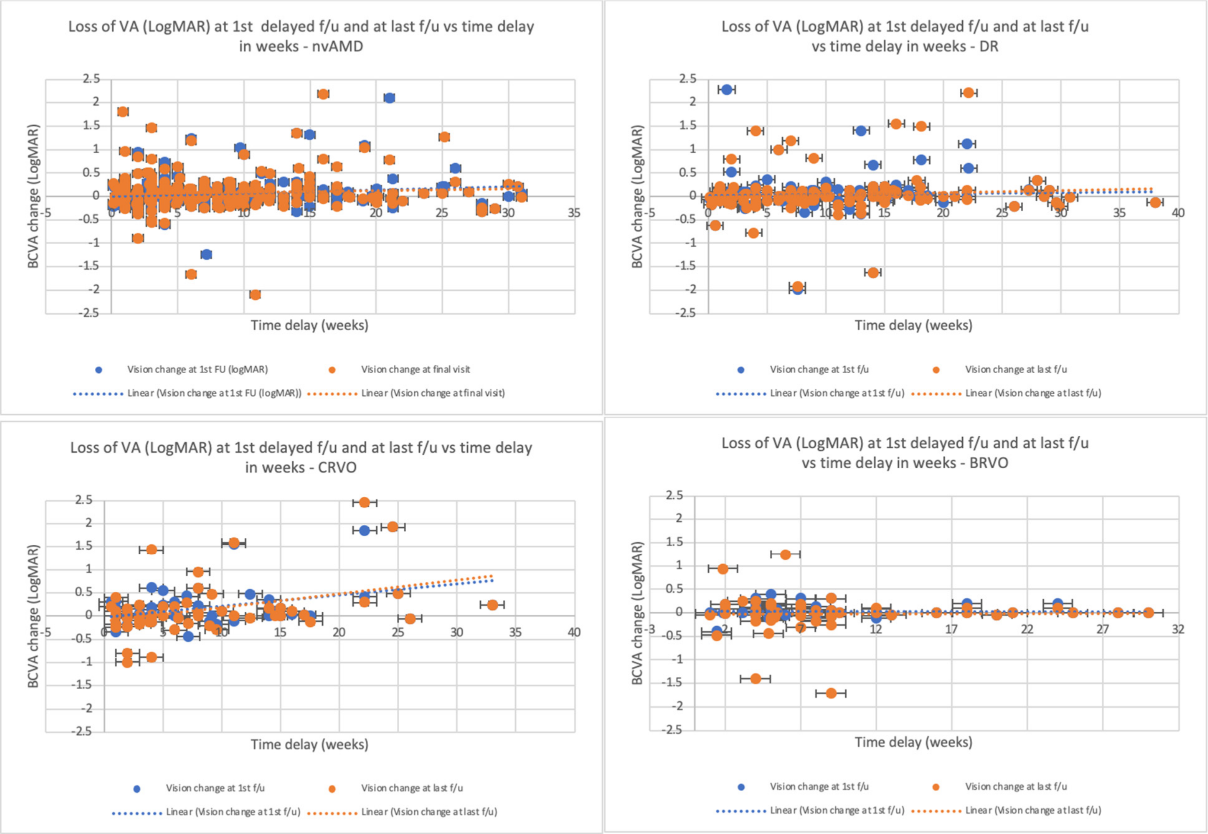

Abbreviations: nvAMD: neovascular Age-related Macular Degeneration; DR: diabetic retinopathy; CRVO: central retinal vein occlusion; BRVO: branch retinal vein occlusion; BCVA: best corrected visual acuity; f/u: follow up; LogMAR: Logarithm of the Minimum Angle of Resolution; SD: standard deviation

* LogMAR of (+) 0.1 equals loss of 5 letters
